# Anatomic Landmarks to Locate the Median Nerve for Safe Wrist Block or Carpal Tunnel Steroid Injection

**Published:** 2019-07-29

**Authors:** Ron Brooks, Amanda Kistler, Saeed Chowdhry, Andrew Swiergosz, Katharina Perlin, Morton L. Kasdan, Bradon J. Wilhelmi

**Affiliations:** Division of Plastic Surgery, University of Louisville, Louisville

**Keywords:** carpal tunnel syndrome, injection, iatrogenic injury, median nerve injury, wrist block

## Abstract

**Introduction:** Carpal tunnel syndrome is the most common entrapment neuropathy involving the upper extremity. As such, various nonoperative techniques have been developed to aid in management of mild to moderate disease, including local steroid injection. However, definitive guidelines for needle/injection location have not been defined, especially in relation to diminishment of iatrogenic injury to the median nerve. **Methods:** A cadaveric study was designed to determine the average width of the median nerve, as well as its location specifically in relation to the palmaris longus (if present), the flexor carpi radialis, and the midpoint of the wrist. All measurements were obtained at the radial tip of the interstyloid line. **Results:** Data demonstrated that the average width of the median nerve was 7.85 mm and that it lies ulnar in location to the palmaris longus (3 mm), as well as the midpoint of the wrist at the radial tip of the interstyloid line (0.43 mm). Furthermore, the distance between the median nerve and the flexor carpi radialis was measured to be 9.57 mm. **Outcomes:** Therefore, injection location should be radial with respect to the palmaris longus and the midline of the wrist. It can be just ulnar to the flexor carpi radialis tendon or between 8 and 10 mm radial to the midpoint of the wrist in order to prevent median nerve injection and direct trauma to the nerve.

Carpal tunnel syndrome (CTS) is the most common entrapment neuropathy involving the upper extremity. While estimates of prevalence and incidence vary widely in literature, data from pooled analyses suggest that the prevalence in the general population is about 1% to 5%, where an increase was demonstrated in populations whose occupations demanded repetitive hand and wrist movements. Greater than 450,000 carpal tunnel release procedures are performed annually in the United States, totaling a cost of $2 billion. Furthermore, CTS was responsible for 1.6% of injuries that required time off work and demonstrated the longest time away from work, only surpassed by automobile accidents, fracture/crush/dislocation, and other traumas.^1^^,^^2^ As the commonality and economic impact of CTS is immense, a multitude of preventive and conservative management techniques have emerged.

The carpal tunnel is a fibro-osseous channel on the anterior portion of the wrist that is located within the concave arch of the carpus and enclosed by the transverse carpal ligament (TCL).^3^ Ten structures traverse this channel including the 4 flexor digitorum superficialis tendons, 4 flexor digitorum profundus tendons, the flexor pollicis longus tendon, and the median nerve (MN). Under typical situations, these structures are able to slide across one another as well as bony protuberances with little friction relating to their synovial sheaths. However, inflammation and edema within the channel can result in compression of tunnel structures, namely, the MN. Specifically, the narrowest part of the tunnel is located at the level of the hook of the hamate with the overlying TCL and is located about 2 to 2.5 cm distal to the origin of the canal. This is the typical location of compression of the MN seen in patients with CTS.^3^

As the MN enters the wrist through the carpal tunnel, it bifurcates into the digital cutaneous branches and the muscular branch. The digital cutaneous branches terminate as 2 common plantar digital nerves that are responsible for motor innervation to the second lumbrical and sensory innervation to the palm and fingers where the thenar motor branch, known as the “million dollar nerve,” innervates the opponens pollicis, the abductor pollicis brevis, and the superficial part of the flexor pollicis brevis before giving rise to the proper palmar digital branch nerves that provide motor innervations to the first lumbrical and sensory innervations to the ulnar side of the hand.^4^ In relation to CTS, both motor and sensory deficits become apparent including myasthenia most noticeably in abduction and opposition of the thumb, which can lead to atrophy of the thenar eminence in advanced stages as well as varying degrees of paresthesia, hypoesthesia, or anesthesia of the volar side of the radial 3½ digits with retention of sensation to the central palm including the thenar eminence.^5^

Current treatment modalities include physical therapy, anti-inflammatory medications, lifestyle modification, splinting, steroids (oral or local), and surgery. Specifically, local injection of steroids not only has been used to treat mild to moderate CTS, with as many as 50% having a good long-term effect for more than 15 months, but also serves as a diagnostic and prognostic tool in the evaluation of potential efficacy of future surgery.^6^^,^^7^ In fact, Green^6^ in “Diagnostic and Therapeutic Value of the Carpal Tunnel Injection” demonstrates a correlation between results of steroid injections with respect to symptom alleviation or resolution and efficacy of future surgery. However, inappropriate needle placement can lead to injury to the medial nerve, the ulnar neurovascular bundle, or the radial artery. In efforts to prevent associated injuries, various techniques have been suggested including injection just radial to the palmaris longus (PL) tendon, in line with the fourth digit, between the PL and flexor carpi radialis (FCR) tendon, between the PL and flexor carpi ulnaris (FCU) tendon, and through the FCR tendon; however, injury prevention has been inconsistent.^8^ One consideration is anatomic variability in the course and branches of the MN. Henry et al in “The Prevalence of Anatomical Variations of the Median Nerve in the Carpal Tunnel: A Systematic Review and Meta-Analysis” classified variations in MN in the hand, including variations in the course of the thenar motor branch, accessory branches of the MN at the distal carpal tunnel, high bifurcation of the MN, and branching of the MN proximal to the carpal tunnel, finding prevalence of 11% to 75%, 4.6%, 2.6%, and 2.3%, respectively.^4^ As such various methods have been established to attempt to standardize injection location. Our aim was to identify a method of local steroid injection that would diminish if not eliminate iatrogenic injury to the MN, especially in the setting of the absence of relevant superficial landmarks such as the PL tendon.

## STUDY DESIGN

**Figure 1:**
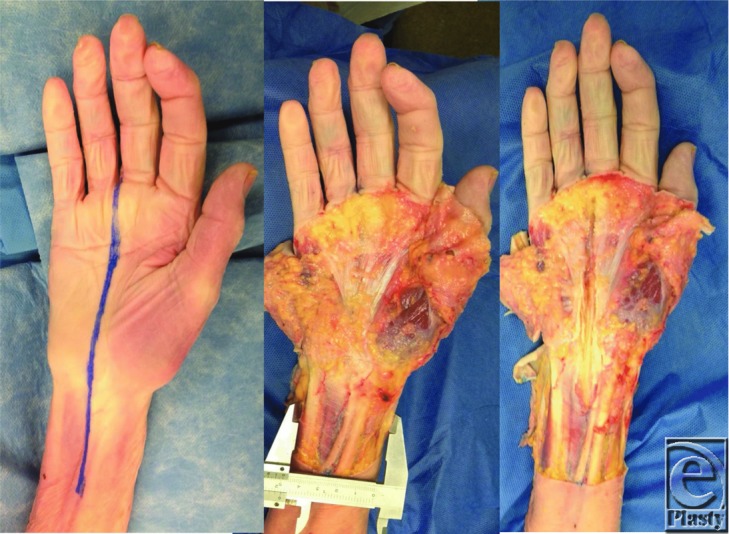
Anatomic dissection with demonstration of the median nerve and palmaris longus near midline.

An anatomic cadaveric study was designed where 7 cadaveric upper extremities were utilized to obtain specific dimensions of the MN as well as its anatomic relationship to other relevant carpal tunnel and non–carpal tunnel components. Loupe magnification (2.5×) was used for the dissection. A longitudinal incision was made from the ulnar aspect of the middle finger, the third web space, to the mid forearm, and relevant anatomic landmarks were identified (Fig 1). Measurements were taken at the radial tip of the interstyloid line and performed with a Vernier side caliper. All measurements were obtained at the level of the radial tip of the interstyloid line. Each of the aforementioned distances were recorded in millimeters and used to calculate mean distances and the standard deviation. These data were extrapolated and relative calculations are demonstrated in Table 1. The prefix of “u” was assigned to indicate measurement or calculation from the ulnar aspect of the anatomic structure. These data were then used to characterize the size of the MN and its anatomic location relative to previously named structures in order to characterize a location for needle placement that would avoid iatrogenic injury to the MN.

## RESULTS

All of the measurements were taken at the level of the radial tip of the interstyloid line. In the 7 dissections, the mean diameter of the MN was 7.85 mm ± 1.8 mm and the distance from the ulnar edge of the MN to the midpoint of the wrist and also the PL tendon was 3.17 mm ± 0.75 mm and 3 mm ± 0.89 mm, respectively. The distance from the ulnar edge of the MN to the ulnar edge of the FCR tendon was 9.57 mm ± 2 mm. These measurements suggest that the MN lies slightly ulnar to the PL tendon and the midpoint of the wrist at the level of the radial tip of the interstyloid line. As a result of these measurements, we propose that the injections be placed just ulnar to the FCR tendon or 8 to 10 mm radial to the midpoint of the wrist to avoid inadvertent direct MN injection and injury.

## DISCUSSION

Inadvertent nerve injection can have devastating functional consequences. Iatrogenic injection-related injury has a 2% incidence according to literature, 3.6% of that specific to the MN. However, these statistics represent injuries to the MN at both the wrist and the cubital fossa near the location of the median cubital vein.^9^ No specific statistics exist for iatrogenic nerve injury of the MN in the location of the carpal tunnel with relation to local steroid injection, as most reported data are in the form of case reports.

Still, research has demonstrated the anatomic, both on the micro and macro scales, effect on the nerve as well as the resultant symptoms. Histological studies have demonstrated that intraneural steroid injection leads to thickened white plaque, which has been referred to a “granuloma,” at both the epineural and perineural levels.^10^ Conduction studies have also demonstrated an increased latency in compound motor action potentials measured to be 4.35 and 4.20 milliseconds at 2 weeks and 6 months postinjection where values greater than 4.20 milliseconds were determined to be abnormal. Perceived sensory changes also diminished over time and were accompanied by a decrease in the latency of the sensory nerve action potentials.^11^ Clinical symptoms present at the time of injection are characterized as a shooting or burning pain in the distribution of the MN and can progress to varying degrees of paresethsias including hypo/hyperesthesia and anesthesia, allodynia, and hypoalgesia as well as decreased motor function.^9^^-^12 It has been suggested that motor function is impacted to a greater degree but that there is a high likelihood of spontaneous recovery with usually a mild deficit.^9^^,^^13^ To avoid injury, one may consider awake injections where a patient provides feedback relating to sensory or motor symptoms, allowing the physician to reposition or remove the needle as well as considering a smaller diameter needle. As a consequence of iatrogenic injection injuries that can lead to permanent deficits, multiple injection locations have been suggested.

Menge et al^8^ in “Carpal Tunnel Injections: A Novel Approach Based on Wrist Width” referenced multiple different injection locations to minimize iatrogenic injury including injection radial to the PL tendon, in line with the fourth digit, between the PL and FCR tendon, between the PL and FCU tendon, and through the FCR tendon with a risk of tendon rupture with direct injection. However, they acknowledged a lack of consistency in injury prevention. They discussed significant variability in the use of superficial landmarks to identify the carpal tunnel as well as injection techniques and advocated using the wrist width for both volar radial and ulnar injection techniques. Specifically, the wrist width was measured at the distal wrist flexion crease from the skin overlying the radial styloid extending to the skin overlying the ulnar styloid, commonly known as the interstyloid line. Findings demonstrated that using 30% to 33% of the wrist width from the radial styloid for volar radial injections demonstrated no direct injection or perforation of the radial artery or MN.^8^ Still, this technique is one of many that are described (Table 2).^8^^,^^14^^-^23 Of note, it is important to mention that injection between the PL and FCR tendon does place the palmar cutaneous branch of the MN at risk of iatrogenic injury, as it typically lies only 8 mm radial to the PL tendon.^24^

**Figure 2:**
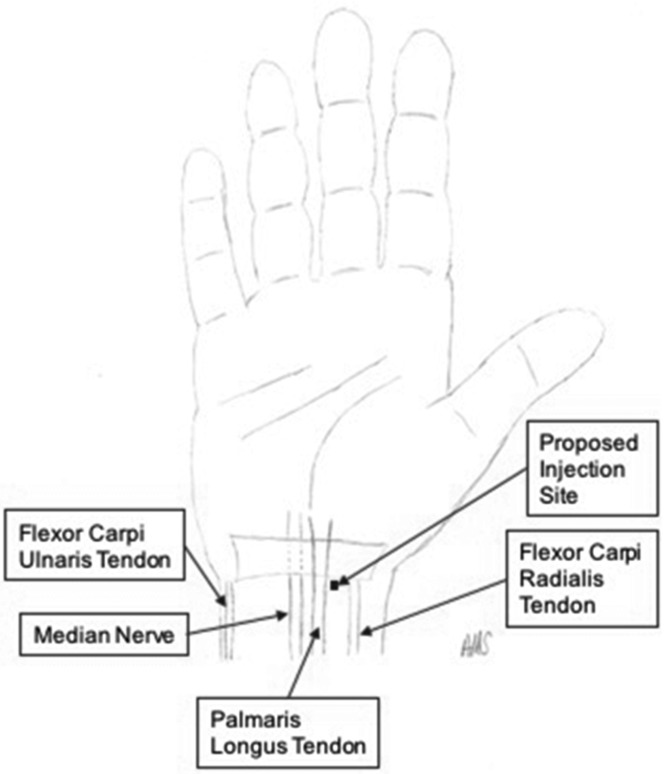
Suggested injection site to avoid iatrogenic injury to the median nerve.

Kim et al^12^ in “Median Nerve Injuries Caused by Carpal Tunnel Injections” also echoed Menge et al in acknowledging the innumerable injection locations (Fig 2). However, they go on to further state that steroid injections should be used sparingly and advocate only the most commonly used approach of needle insertion just ulnar to the PL tendon at the wrist crease as well as ultrasound guidance, if possible.^12^ Nonetheless, they note that iatrogenic nerve injection does occur and promote both surgical and nonsurgical management. Nonsurgical management consists of physical therapy, activity modification, splinting, and anti-inflammatory medications, methods that are attempted with less advanced CTS. If improvement is not observed within 3 months or if there is severe neuromotor loss or severe debilitating pain, they recommend consideration of surgical correction including neurolysis, resection with graft repair, and/or debridement.^12^ With a clear lack of defined injection location, our study used anatomic measurements to identify a location that would be free of iatrogenic injection injuries.

Using 7 cadavers from the fresh tissue laboratory at the University of Louisville, we were able to identify a safe location for steroid injection that would prevent intraneural injection. As various injection techniques exist, as discussed previously, we used common landmarks such as the PL and FCR tendons. As research supports, the MN is typically located ulnar to the PL tendon, which was supported by our data. Specifically, our data demonstrated an average distance from the PL to the MN of 3 mm, data support up to 13 mm, where all cadavers demonstrated an ulnar located MN with respect to the PL, again where research demonstrates that ulnar location in 88% of hands.^14^ Similarly, our finding that the MN is typically located ulnar to the midpoint of the wrist by 0.43 mm led to our assertion that injection to the PL tendon would prevent iatrogenic MN injury. Furthermore, our data used the FCR tendon as the radial most measurement with relation to the MN as it is located just outside of the carpal tunnel and its tendon can be easily palpated. The distance calculated from the MN to the FCR was 9.57 mm. As the average width of the MN in our study was 7.85 mm and the PL tendon was 6.25 mm, we were able to combine our data to recommend ideal injection location ulnar to the FCR tendon or between 8 and 10 mm radial to the midpoint of the wrist.

## CONCLUSION

Injection-related MN injury can cause lasting and debilitating sensory motor deficits. As such, precision in needle placement for injection is paramount. Many studies exist with respect to injection location, but a lack of consistent data related to injury prevention exists. On the basis of anatomic measurements, we have concluded that the safest injection location site is located on the ulnar aspect of the FCR tendon or 8 to 10 mm radial to the midpoint of the wrist while maintaining an injection site that is radial to the PL tendon.

## Figures and Tables

**Table 1:** Calculated anatomic measurements (mm)*

Average width of the MN
Average width of the PL
Average distance between the uMN to uPL
Average distance between uMN to MP
Average distance between uMN to uFCR

*MN indicates median nerve; PL, palmaris longus; u, measurement or calculation from the ulnar aspect of the anatomic structure; MP, midpoint of the wrist; FCR, flexor carpi radialis.

**Table 2:** Recommended anatomic locations for prevention of iatrogenic median nerve injury caused by injection*

Author(s)	Year of publication	Anatomic location
Gelberman et al^22^	1980	1 cm proximal to the distal wrist crease between the PL and FCR tendons with 45°-60° angle
Green^6^	1984	Ulnar to PL
Linskey and Segal^17^	1990	Ulnar to PL tendon at the distal wrist crease, if present. If not present, injection in line with the fourth digit
Frederick et al^16^	1992	Midway between PL tendon and FCU tendon just proximal to the wrist crease. If PL not present, inject in line with the FDS tendon of the fourth digit
Kasten and Louis^15^	1996	Distal and dorsal direction starting on the radial border of the pisiform toward the mid portion of the carpal tunnel
Dammers et al^21^	1999	Volar side of the forearm between PL tendon and FCR tendon, 4 cm proximal to the wrist crease
Graham et al^23^	2004	Just ulnar to FCR
Dubert and Racasan^20^	2006	Through FCR tendon, 1 cm proximal to the wrist crease with the needle angled at 45° toward the medial edge
MacLennan et al^19^	2009	Ulnar to FCR tendon at the wrist crease
Kamanli et al^18^	2011	Ulnar to PL tendon distal to the wrist crease
Menge et al^8^	2016	30%-33% of wrist width from the radial styloid

*PL indicates palmaris longus; FCR, flexor carpi radialis; FCU, flexor carpi ulnaris; FDS, flexor digitorum superficialis.
